# Upregulation of CPNE7 in mesenchymal stromal cells promotes oral squamous cell carcinoma metastasis through the NF-κB pathway

**DOI:** 10.1038/s41420-021-00684-w

**Published:** 2021-10-14

**Authors:** Xiaoli Ji, Tianyong Sun, Shang Xie, Hua Qian, lixiang Song, lihua Wang, Hongwei Liu, Qiang Feng

**Affiliations:** 1 Department of Stomatology, Jinan Central Hospital, Cheeloo College of Medicine, Shandong University, No.105 Jiefang Road, Jinan, 250013 Shandong China; 2 Department of Oral Mucosal Diseases, School and Hospital of Stomatology, Cheeloo College of Medicine, Shandong University & Shandong Key Laboratory of Oral Tissue Regeneration & Shandong Engineering Laboratory for Dental Materials and Oral Tissue Regeneration, No.44-1 Wenhua Road West, Jinan, 250012 Shandong China; 3 Department of Human Microbiome, School and Hospital of Stomatology, Cheeloo College of Medicine, Shandong University & Shandong Provincial Key Laboratory of Oral Tissue Regeneration & Shandong Engineering Laboratory for Dental Materials and Oral Tissue Regeneration, No.44-1 Wenhua Road West, Jinan, 250012 Shandong China; 4 Department of Oral and Maxillofacial Surgery, Peking University School and Hospital of Stomatology, 22 Zhongguancun South Avenue, Haidian, Beijing, 100081 China; 5 Department of Stomatology, The Second Hospital of Shandong University, No. 247 Beiyuan Road, Jinan, 250033 China; 6 Department of Pediatric Dentistry, School and Hospital of Stomatology, Cheeloo College of Medicine, Shandong University & Shandong Key Laboratory of Oral Tissue Regeneration & Shandong Engineering Laboratory for Dental Materials and Oral Tissue Regeneration, No.44-1 Wenhua Road West, Jinan, 250012 Shandong China; 7 Department of Oral Medicine, Peking University School and Hospital of Stomatology, 22 Zhongguancun South Avenue, Haidian, Beijing, 100081 China; 8 NHC Key Laboratory of Otorhinolaryngology (Shandong University), No.44-1 Wenhua Road West, Jinan, Shandong China 250012

**Keywords:** Oral cancer detection, Oral cancer detection

## Abstract

A remarkable shift in Mesenchymal stromal cells (MSCs) plays an important role in cancer metastasis, but the molecular mechanism is still unclear. CPNE7, a calcium-dependent phospholipid-binding protein, mediates signal transduction and metastasis in many tumours. Here, we demonstrated that MSCs derived from OSCC (OSCC-MSCs) promoted the metastasis of OSCC cells by transwell assay and animal models through epithelial to mesenchymal transition (EMT) (p < 0.05). RNA-sequencing, ELISA, neutralizing antibody and CXCR2 inhibitor assay confirmed that CXCL8 secreted by OSCC-MSCs was associated with the upregulated expression of CPNE7 by immunohistochemical and western blotting (p < 0.05). This is mechanistically linked to the activation of CPNE7 to NF-κB pathway-induced metastasis, including phosphorylated p65 and IκBa. CPNE7 silencing inhibited metastatic abilities and the expression of CXCL8, phosphorylated p65, IκBa, and p65 nuclear translocation by western blotting and immunofluorescence, while CPNE7 overexpression markedly promoted these events (*p* < 0.05). We also identified that Nucleolin could be bind CPNE7 and IκBa by co-immunoprecipitation. Together, our results suggest that upregulation of CPNE7 in MSCs interacted with surface receptor -Nucleolin and then combined with IκBa to promoted phosphorylated IκBa and p65 nuclear translocation to active NF-κB pathway, and then regulates CXCL8 secretion to promote the metastasis of OSCC cells. Therefore, CPNE7 in MSCs could be promising therapeutic targets in OSCC.

## Introduction

Oral squamous cell carcinoma (OSCC) accounts for more than 90% of oral cancer and is one of the most prevalent cancers worldwide [1–3]. Despite advancements in treatment, metastasis is still the main cause of OSCC-related death [2]. In this process, tumour cells have to acquire an epithelial-mesenchymal transformation (EMT) phenotype, by which epithelial cells gain mesenchymal properties [4]. The earliest stage of EMT does not take place in metastatic progression but in pre-neoplastic lesions [5, 6]. Oral leukoplakia (OLK), with an incidence of 4.1% worldwide, is the most common pre-neoplastic lesion of OSCC [7, 8]. Currently, the challenge is to prevent the metastasis of OSCC in the pre-neoplastic lesion, but the related mechanism remains unclear.

Mesenchymal stromal cells (MSCs) are critical components of the tumour microenvironment (TME) and play a crucial role in the metastasis of tumours [9–11]. MSCs, as progenitors of stromal cells, have been isolated from different stages of normal tissues, pre-neoplastic lesions, and cancer tissues [12–15]. In normal tissue, MSCs are in a quiescent state to maintain tissue architecture [16]. With the initiation of tumorigenesis, MSCs gradually begin to transition into a determined reprogrammed state accompanied by aberrant mutations in tumour cells [9, 10]. The TME provides a niche for MSCs and tumour cells to modify or communicate with each other, in which confers a pro-migratory state of the TME [14, 15]. Therefore, an increasing number of studies have focused on the question of whether pre-neoplastic or tumour-associated MSCs have distinct characteristics with a unique gene expression profile in a paracrine manner [17–19]. Studies have shown that MSCs derived from cervical intraepithelial neoplasia (precancerous lesion of the uterine cervix) can secrete several cytokines to sustain an anti-tumour microenvironment and block the progression of tumours [19], breast cancer-derived MSCs can alter a significantly dysregulated secretion profile, and breast tumour cells exhibit a more aggressive phenotype through the activation of epithelial-to-mesenchymal transition in vitro and in vivo [20]. To date, most related research has reported the specific cytokines secreted by tumour-associated MSCs that affect the progression of tumours, but the detailed mechanism of MSC-secreted cytokines in tumour progression is scarcely reported, although it is important to modify the TME through MSCs to prevent tumorigenesis.

In this study, we investigated the influence of OLK-MSCs (MSCs derived from OLK) and OSCC-MSCs (MSCs derived from OSCC) on OSCC cell migration and invasion through EMT. We performed RNA-sequencing to identify the key cytokines localized to specific pathways in MSCs that explain the pro-metastatic effects of OSCC-MSCs, and a neutralizing antibody, receptor inhibitor, and pathway inhibitor were used to verify the results. In addition, we also identified the upstream regulator of this pathway in MSCs, and the results were confirmed by gene overexpression, knockdown experiments, and co-immunoprecipitation.

### Materials and Methods

#### Tumour cell line culture

OSCC cell lines CAL27(maintained in our lab) and WSU-HN6 (a kind gift from Professor Yixiang Wang at Peking University) were cultured in Dulbecco’s modified Eagle’s medium(DMEM,HyClone,Kansas,USA) supplemented with 10% fetal bovine serum (FBS) and 1% penicillin-streptomycin (Gibco, Washington, USA) in a humidified incubator at 37 °C with 5% CO_2_.

#### Isolation of OLK-MSCs and OSCC-MSCs

Oral mucosal tissues were obtained from 16 donors (8 were pathologically confirmed as tongue OLK [21] and 8 were oral (tongue)squamous cell carcinoma [22]) at the School and Hospital of Stomatology, Cheeloo College of Medicine, Shandong University. Tissues were treated by dispase (2 mg/ml, Sigma-Aldrich, Darmstadt, Germany) washed in PBS, and then cut into 1 mm^3^-sized pieces and cultured in a T25 flask with α-minimum essential medium (α-MEM; Gibco, Washington, USA) containing 10% fetal bovine serum (FBS; Gibco, Washington, USA) and 1% penicillin-streptomycin at 37 °C in a humidified 5% CO_2_ atmosphere to obtain MSCs (passage 3–5 were used for sequent experiment). All patients signed the informed consent form. This study was approved by the ethics committee of the School and Hospital of Stomatology, Cheeloo College of Medicine, Shandong University (NO. GR201804).

### Identification of OLK-MSCs and OSCC-MSCs

The methods for the identification of MSCs followed the Minimal Criteria for Defining Multipotent Mesenchymal Stromal Cells [23]. OLK-MSCs and OSCC-MSCs were treated with PE-conjugated human STRO-1, CD105 (BD Biosciences, California, USA), and CD45 and FITC-conjugated human CD29, CD73, CD90, and CD34 (BioLegend, California, USA). The control was isotype-matched control IgG or IgM. The osteogenic and adipogenic potential of OLK-MSCs and OSCC-MSCs was identified by the related induction media.

### Generation of conditioned medium from MSCs

OLK-MSCs, OSCC-MSCs, CPNE7-targeting siRNA (si-CPNE7), scrambled non-targeting control siRNA (si-control), lentivirus-CPNE7-GFP (lv-CPNE7) and lentivirus-control-GFP (lv-control) (the number of the above MSCs = 1.375 × 10^6^) were plated at in complete medium (13.75 ml) in a 10 cm plate for 5 days to generate conditioned medium (CM). The CM was collected through a 0.2 μm filter and stored at −80 °C.

### Transwell migration and invasion assay

These assays were measured in 24-well Transwell inserts (24well, 8.0 μm, Corning, NY, USA) with or without Matrigel (300 µg/ml, Corning, NY, USA). OLK-MSCs and OSCC-MSCs (5 × 10^4^ for 5 days), CM derived from OLK-MSCs or OSCC-MSCs, serum-free CM derived from OLK-MSCs or OSCC-MSCs (added 10% FBS), CM of OSCC-MSCs alone or in the presence of a human CXCL8 neutralizing antibody (0.4 μg/ml, R&D Systems, Minnesota, USA) (or control IgG (negative control, Santa-Cruz, California, USA)), medium added with 2000 pg/ml and 30000 pg/ml CXCL8 (PeproTech GmbH, Hamburg, Germany), CM from si-CPNE7 and si-control or CM from lv-CPNE7 and lv-control (the volume of CM = 500 ul) were added to the lower chamber (24 well plate). CAL27 cells (10^5^) or WSU-HN6 cells (5 × 10^4^) (or treated with 400 nM CXCR2 inhibitor SB225002 (TargetMol, Boston, MA, USA) or 0.001% DMSO) in serum-free medium were added to the upper Transwell inserts (24well, 8.0 μm, Corning, NY, USA). After incubation at 37 °C for 18 h, a cotton swab was used to remove the cells of the upper chamber and the invaded cancer cell on the lower side of the membrane were fixed with 4% paraformaldehyde and stained with 0.1% crystal violet for 30 min, respectively. The number of invaded cancer cells on the lower side of the membrane was counted with a light microscope in three randomly selected fields at 100× and 400× magnification. The average number of each field was calculated.

### Animals’ models

Four-week-old female BALB/c nude mice (SPF(Beijing)BIOTECHNOLOGY Co., Ltd.) were maintained in a sterile environment. 10^6^ OLK-MSCs+10^6^ WSU-HN6 or 10^6^ OSCC-MSCs+10^6^ WSU-HN6 in PBS injected into the tail vein of BALB/c nude mice. All mice were killed by 4 weeks after injection. The metastatic nodules in the lung were recorded and stained with H&E. The animal experiments were approved by the ethics committee of the School and Hospital of Stomatology, Cheeloo College of Medicine, Shandong University (NO. GR201804).

### Western blot assay

Protein was obtained with RIPA buffer. Equal amounts of proteins were resolved by 10% SDS-PAGE [24] and immunoblotted with primary antibodies against the following antigens overnight: E-cadherin, alpha E catenin, Vimentin, N-cadherin, GFP (Proteintech, Wuhan, China), p-p65, total-p65 (Abcam, Cambridge, England), p-Iκβα, total-Iκβα (CST, California, USA), CPNE7 (GeneTex, Texas, USA), and GAPDH (Affinity, Ohio, USA), incubated with the HRP-conjugated secondary antibodies (Affinity, Ohio, USA). Positive immunoreactive bands were detected by enhanced chemiluminescence (ECL, Amersham Pharmacia Biotech, USA) reaction and normalized by GAPDH with quantity one.

### RNA-sequencing analysis

Total RNA was extracted from OLK-MSCs and OSCC-MSCs (3 cases in each group) with Trizol (Thermo Fisher Scientific) and total RNA was isolated according to the manufacturer’s protocol. The differentially expressed genes were identified with edgeR (3.30.3) (*p* < 0.05, ┃fold change(FC)┃ > 2), and KEGG pathway analysis was performed (Database string C).

### Retrieval of The Cancer Genome Atlas (TCGA) data

TCGA expression data were retrieved from UALCAN (http://ualcan.path.uab.edu/). TCGA datasets were stratified into normal and primary tumours.

### Enzyme-linked immunosorbent assay (ELISA)

The CM of OLK-MSCs, OSCC-MSCs, si-CPNE7, si-control, lv-CPNE7, and lv-control was obtained as described above. The concentration of CXCL8 was measured by ELISA (BioLegend, California, USA) according to the manufacturer’s instructions.

### Immunohistochemistry (IHC) assays

Immunohistochemical staining was routinely performed according to a standard protocol. The sample (42 OLK, 62 OSCC) were obtained from the Department of Oral Pathology (School and Hospital of Stomatology, Cheeloo College of Medicine, Shandong University). Histopathological evaluation of samples was performed by two pathologists. Samples were stained with a CPNE7 antibody (Bioss, Beijing, China) [25]. For the immunohistochemical scoring of CPNE7, the staining CPNE7-intensity was scored as 0 (negative), 1 (low Positive), 2 (positive), and 3 (high positive) [26] and the proportions of CPNE7-positive cells were counted using the Fiji open-source image processing program (ImageJ software, USA) [26]. Both the CPNE7-staining intensity and the proportions of CPNE7-positive cells were taken as the average value of three random optical fields in the epithelial and connective tissue, respectively [26, 27]. The multiplication for the above intensity and proportion was used to evaluate the expression level of CPNE7 [26, 27].

### Immunofluorescence histochemistry

Tissues collected from patients were stored at −80 °C and cut into 4 μm sections. Alternatively, MSCs were seeded in 12-well plates with sections overnight and fixed with 4% paraformaldehyde. Sections were incubated with 3.0% hydrogen peroxide and then blocked in 5.0% bovine serum albumin. The sections were incubated at 4 °C overnight with antibodies against CPNE7 (Bioss, Beijing, China), STRO-1 (Santa -Cruz, California, USA), or p65 (CST, California, USA), Nucleolin. Subsequently, incubation with Alexa Fluor 488/594-labelled goat anti-mouse/rabbit IgG (Abbkine, California, USA) was for 1 h at 37 °C in the dark.

### Small interfering RNA (siRNA) transfection

si-CPNE7 and si-control were obtained from GenePharma (Suzhou, China). The sequences were as follows: CPNE7 sense 5′-CCGGGAAAGCCUCUCAAUATT-3′, CPNE7 antisense 5′-UAUUGAGAGGCUUUCCCGGTT-3′, control sense 5′-UUCUCCGAACGUACGUTT-3′, and control antisense 5′-ACGUGACACGUUCGGAGAATT-3′. 2 × 10^5^ cells were seeded in each well of a 6-well plate overnight. 10 nM siRNA was diluted in Opti-MEM^®^ I Reduced Serum Medium without serum and mixed with Lipofectamine 2000 RNAiMAX Reagent (Thermo Fisher Scientific, New York, USA). The mixture was added to each well and incubated for 6 h.

### Lentivirus and transfection

The lv-CPNE7 and lv-control were constructed by Gene Pharma (Suzhou, China). OLK-MSCs were incubated with a medium containing lentivirus and polybrene. After 24 h, the medium was changed, and incubation proceeded for 48 h.

### Co-IP assay

lv-CPNE7 transfected OLK-MSCs’ lysates were treated by NP-40 buffer. The lysates were incubated at 4 °C overnight with GFP or total total-Iκβα, Nucleolin and normal IgG (Santa, California, USA) with rotation. Protein A/G PLUS agarose beads (Santa, California, USA) were added and incubated at 4 °C for 1 h with rotation. The complexes were released from the beads by boiling in an SDS-PAGE sample buffer. The immunoprecipitates were analysed by western blotting with GFP, CPNE7, and total-Iκβα, Nucleolin.

### Statistical analysis

All experiments were repeated 3 times and the data are presented as mean ± SD. Comparison between two groups were performed using paired and two-tailed Student’s *t*-test. The considered statistically significant was *p* ≤ 0.05. The data were analysed using SPSS software version 17.0.

## Results

### Identification of OLK-MSCs and OSCC-MSCs

To verify the characteristics of MSCs, a panel of antibodies against MSC surface antigens was used to label OLK-MSCs and OSCC-MSCs and analyse the cells by flow cytometry. The results showed that OLK-MSCs and OSCC-MSCs were positive for CD29, CD73, CD90, CD105, and STRO-1 and negative for CD34 and CD45 (Supplementary Fig. 1A). Multilineage differentiation experiments showed that OLK-MSCs and OSCC-MSCs could form mineralized nodules under osteoinductive conditions (Supplementary Fig. 1B), form oil droplets upon adipogenic induction (Supplementary Fig. 1C), and chondrogenesis (Supplementary Fig. 1D), respectively.

### OSCC-MSCs promote cancer metastasis by EMT

**Fig. 1:**
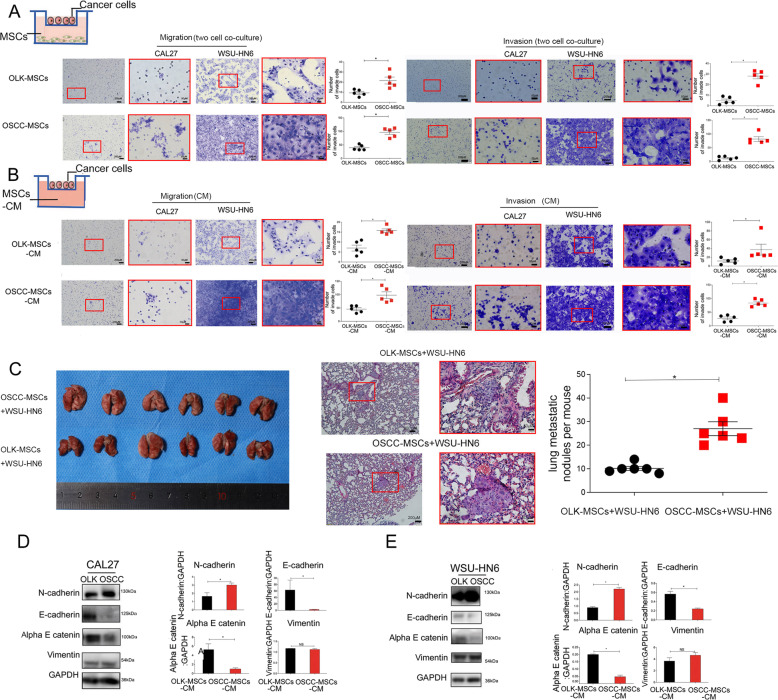
OSCC-MSCs promoted the metastasis of OSCC cell lines. Transwell assays were performed to detect tumour (CAL27 and WSU-HN6) cell migration and invasion through indirect cell-cell contact co-culture (**A**) and conditioned medium (**B**) from OLK-MSCs and OSCC-MSCs (*n* = 5). **C** OLK-MSCs or OSCC-MSCs (10^6^) were co-injected WSU-HN6 (10^6^) into BALB/c mice via tail vein (*n* = 6). Metastasis lung tumor nodules were counted after 28 days. OSCC-MSCs promote the metastasis of CAL27 (**C**) and WSU-HN6 cells (**D**) through epithelial‐mesenchymal transition (EMT)-related proteins (E‐cadherin, N‐cadherin, alpha E catenin, and Vimentin). The tests were repeated three times. Data were expressed as means ± SD. *p < 0.05.

To evaluate the effect of OLK-MSCs and OSCC-MSCs on the migration and invasion of OSCC cells, we constructed a two-cell co-culture system using a transwell chamber and showed that co-culture with OSCC-MSCs significantly increased the number of migrated and invasive CAL27 and WSU-HN6 cells (*p* < 0.05) compared with OLK-MSC co-culture (Fig. 1A). To further verify the metastasis-promoting effect of OSCC-MSCs, CM from OLK-MSCs and OSCC-MSCs was obtained and used to treat CAL27 and WSU-HN6 cells in transwell assays. The results showed that the migration capability of CAL27 and WSU-HN6 cells was enhanced by OSCC-MSC-CM compared to OLK-MSC-CM (*p* < 0.05) (Fig. 1B). Furthermore, the metastasis-promoting effect of OSCC-MSC-CM was like that observed in the OSCC-MSC-cancer cell co-culture system. To eliminate the effect of serum consumption during generation of conditioned medium, CM was acquired in no FBS medium of OLK-MSCs and OSCC-MSCs for 5 days. The results showed that pro-metastasis of OSCC-MSCs was also confirmed and not due to the serum deprivation (Supplementary Fig. 2).

We next tested whether OSCC-MSCs can effectively promote OSCC cell in vivo, 10^6^ OLK-MSCs and 10^6^ WSU-HN6 or 10^6^ OSCC-MSCs and 10^6^ WSU-HN6 were injected via the tail vein of nude mice. Fig 1C indicated that typical cancer tissues changes within the lungs staining with H&E in two groups and the group of OSCC-MSCs + WSU-HN6 revealed a remarkable increase in the number of metastatic nodules in the lung when compared with OLK-MSCs (*p* < 0.05). So, the pro-metastatic effect of OSCC-MSCs was be showed.

The expression of E-cadherin, alpha E catenin (epithelial markers), N-cadherin, and Vimentin (epithelial markers) was shown that the levels of E-cadherin and alpha E catenin decreased significantly in CAL27 and WSU-HN6 cells treated with OSCC-MSC-CM compared to the OLK-MSC-CM group (*p* < 0.05), while the expression of N-cadherin notably increased when CAL27 and WSU-HN6 cells were treated with OSCC-MSC-CM in comparison with OLK-MSC-CM (*p* < 0.05) (Fig. 1D, E). No differences in Vimentin were detected between the two groups (*p* > 0.05) (Fig. 1 D, E).

### OLK-MSCs and OSCC-MSCs display different gene expression profiles

**Fig. 2:**
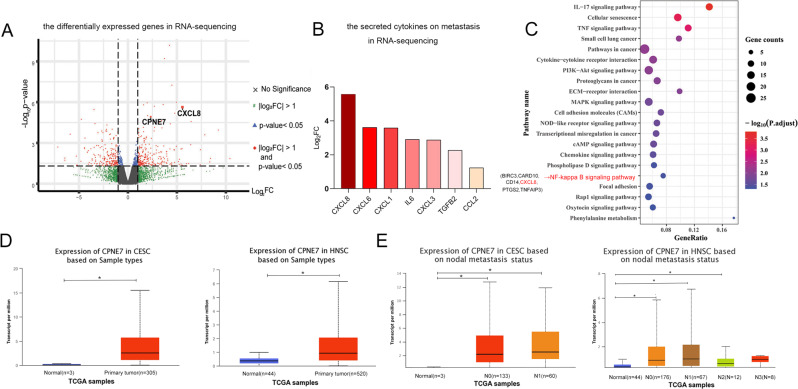
CPNE7 regulates CXCL8 secretion through NF-κB pathway in RNA-sequencing analysis. **A** volcano plot presenting all of the differentially expressed genes between OLK-MSCs and OSCC-MSCs (┃fold change (FC)┃ > 2, *p* - value<0.05). **B** The log_2_FC of the secreted cytokines on metastasis. **C** KEGG pathway analysis between OLK-MSCs and OSCC-MSCs (*p* < 0.05). **D** Expression of CPNE7 in Cervical squamous cell carcinoma (CESC) and Head and Neck squamous cell carcinoma (HNSC) according to TCGA database. **E** Expression of CPNE7 in CESC and HNSC based on nodal metastasis status. N0: No regional lymph node metastasis. N1: Metastases in 1 to 3 axillary lymph nodes. N2: Metastases in 4 to 9 axillary lymph nodes. N3: Metastases in 10 or more axillary lymph nodes. *p < 0.05.

To explore the cytokines involved in OSCC cell metastasis, RNA-sequencing (SRA data (PRJNA665945)) was conducted and showed 454 differentially expressed genes (|log_2_FC | >1, *p* < 0.05):247 genes were upregulated and 207 downregulated in OSCC-MSCs when compared with OSCC-MSCs (Fig. 2A). Figure 2B showed the fold change of the differential genes secreted by MSCs were related to metastasis. And fold change of secreted CXCL8-chemokines was the greatest (upregulated significantly) [27–30]. KEGG analysis showed that several pathways associated with cancer metastasis were activated (*p* < 0.05) (Fig. 2C). CXCL8 has also annotated the above pathway especially the IL-17 signaling pathway, NOD-like receptor signaling pathway, NF-κB pathway, Phospholipase D signaling pathway (Fig. 2C) [31–33]. NF-κB pathway (BIRC3, CARD10, CD14, CXCL8, PTGS2, TNFAIP3) is the centre of the above pathway [3, 34, 35]. Additionally, TCGA data showed that CPNE7 expression in primary tumours was significantly upregulated compared to that in normal tissues in Cervical squamous cell carcinoma (CESC) and Head and Neck squamous cell carcinoma (HNSC) (*p* ≤ 0.05) (Fig. 2D) and could promote nodal metastasis (Fig. 2E). According to the data of RNA- sequencing analysis, CPNE7 in MSCs (the upregulated differential gene) (Fig. 2A) was predicted as a potential regulator of the NF-κB pathway [36] to promote the metastasis of tumour cells.

### OSCC-MSCs secrete more CXCL8, which is regulated by NF-κB pathways

**Fig. 3:**
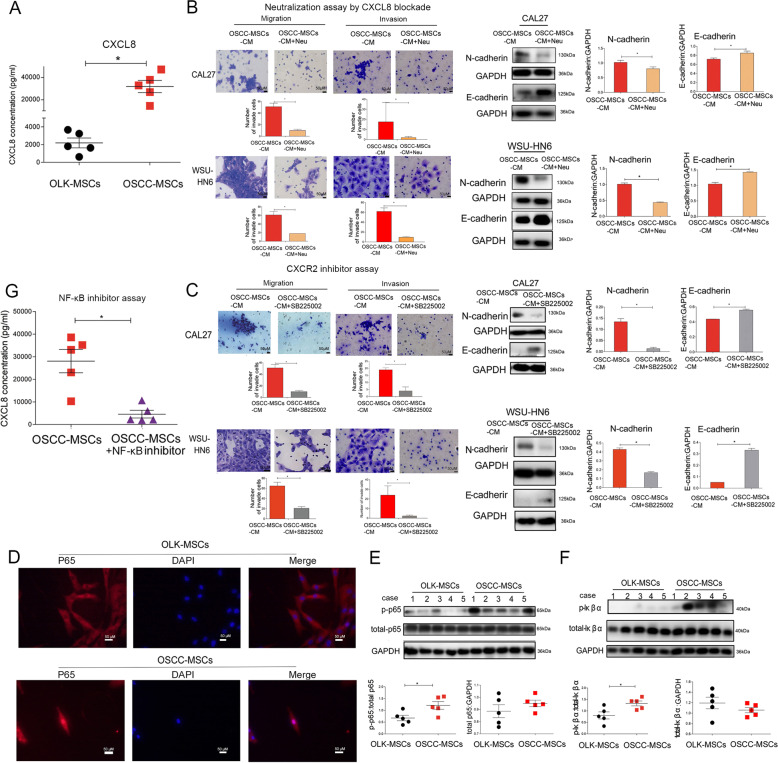
Overexpression CXCL8 secretion regulated by NF-κB pathway in OSCC-MSCs. **A** The level of secreted CXCL8 was observed in the conditioned medium of OLK-MSCs and OSCC-MSCs (*n* = 5). Neutralizing antibody (CXCL8) (**B**) and CXCR2 inhibitor (SB225002) (**C**) inhibited OSCC-CM-induced OSCC cell line metastasis through EMT -related proteins. **D** Representative immunofluorescence images of the translocation of p65 in OLK-MSCs and OSCC-MSCs. Scale bars = 50 µm. Western blotting was used to measure the differential expression of p65 (**E**) and IκBα (**F**) (NF-κB pathway) in OLK-MSCs (*n* = 5) and OSCC-MSCs (*n* = 5). **G** The NF-κB inhibitor suppressed the secretion of CXCL8 by Elisa. The tests were repeated three times. Data were expressed as means ± SD. *p < 0.05.

To validated the results of RNA-sequencing, ELISA showed that compared to OLK-MSCs, secreted CXCL8 was also significantly elevated in OSCC-MSCs (*n* = 5, *p* < 0.05) (Fig. 3A). Additionally, to further determine the metastasis-promoting effect of CXCL8 on OSCC cells, we inhibited the effect of CXCL8 with a neutralizing anti-CXCL8 antibody and an inhibitor of the CXCL8 receptor CXCR2 (SB225002). The results showed that the metastatic effect of OSCC-MSC-CM was significantly inhibited (Fig. 3B, 3C), while E-cadherin expression increased and N-cadherin expression in CAL27 and WSU-HN6 cells decreased (Fig. 3B, 3C). Additionally, to stimulate the microenvironments of OLK-MSCs and OSCC-MSCs, different concentrations of CXCL8 (2000pg/ml and 30,000 pg/ml) were used by migration and invasion assay. The results were like CXCL8 blockade or CXCR2 inhibitor assay (Supplementary Fig. 3).

To confirm the regulation of NF-κB on CXCL8 secretion, p65 was released and translocated into the nucleus in OSCC-MSCS and only found in the cytoplasm of OLK-MSCs by the immunofluorescence assay which confirm NF-κB pathway activation (Fig. 3D). In order to further support the above conclusion, the additional Western blotting analysis showed that phosphorylated p65 levels were increased in OSCC-MSCs of the different individuals (*n* = 5) compared with OLK-MSCs (*n* = 5) (*p* < 0.05) (Fig. 3E). Furthermore, phosphorylated IκBα in the cytoplasm, leading to its ubiquitination and subsequent degradation, could promote p65 is released, translocate to the nucleus, and regulate the expression of genes [37, 38]. Therefore, we detected that OSCC-MSCs (*n* = 5) expressed significantly higher level of phosphorylated Iκßα levels than OLK-MSCs (*n* = 5) (*p* < 0.05) (Fig. 3F). For the further demonstration of the NF-κB pathway regulation of CXCL8, OSCC-MSCs were treated with NF-κB inhibitor (BAY 11-117802), and results demonstrated that CXCL8 secretion was partially reduced (Fig. 3G).

### CPNE7 is upregulated in OSCC tissue and OSCC-MSCs

**Fig. 4:**
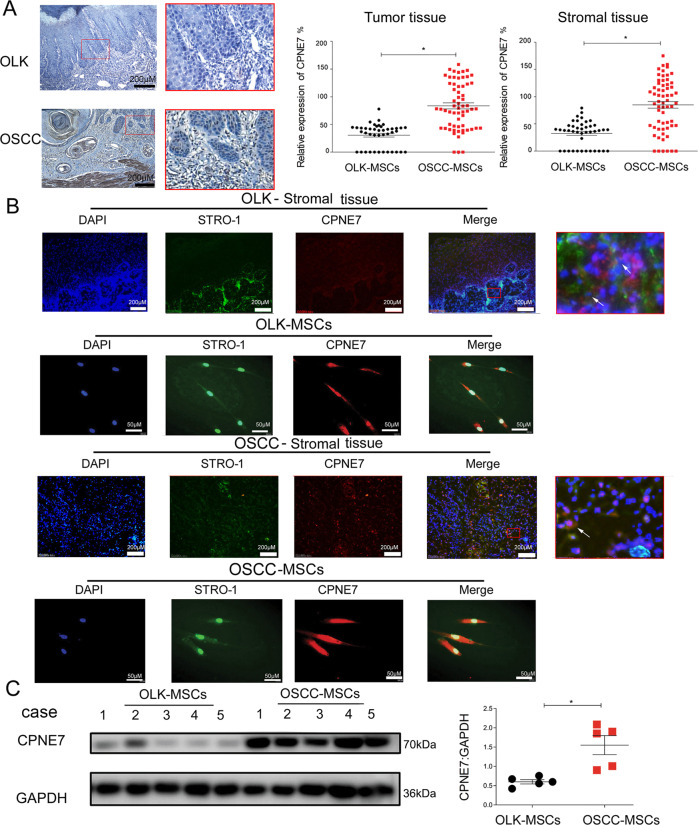
Higher expression of CPNE7 in tissue and MSCs of OSCC than OLK. **A** Representative immunohistochemical images of CPNE7 expression levels in OLK and OSCC tissues. **B** Immunofluorescence detection of the expression of CPNE7 and the MSC marker STRO-1 in tissue and MSCs. The arrow indicated the co-expression of CPNE and STRO-1 in the same cell of tissue. **C** Western blotting was performed to detect the expression of CPNE7 between OLK-MSCs and OSCC-MSCs (*n* = 5). The tests were repeated three times. Data were expressed as means ± SD. *p < 0.05.

CPNE7 was predicted as a potential regulator of the NF-κB pathway by KEGG pathway annotation and RNA-sequencing analysis [36]. To explore the expression level of CPNE7, we performed immunohistochemically staining in clinical tissue. As shown in Fig. 4A, the expression of CPNE7 was detected in the nucleus and cytoplasm and was significantly upregulated in the total tissue or stromal tissue of OSCC compared to OLK. Moreover, immunofluorescent staining showed that CPNE7 and STRO-1 were co-expressed in stromal tissue and MSCs (OLK and OSCC) (Fig. 4B). In addition, we observed an increase in CPNE7 expression in OSCC-MSCs compared with OLK-MSCs (Fig. 4C).

### Upregulation of CPNE7 in OSCC-MSCs promote cancer metastasis by EMT

**Fig. 5:**
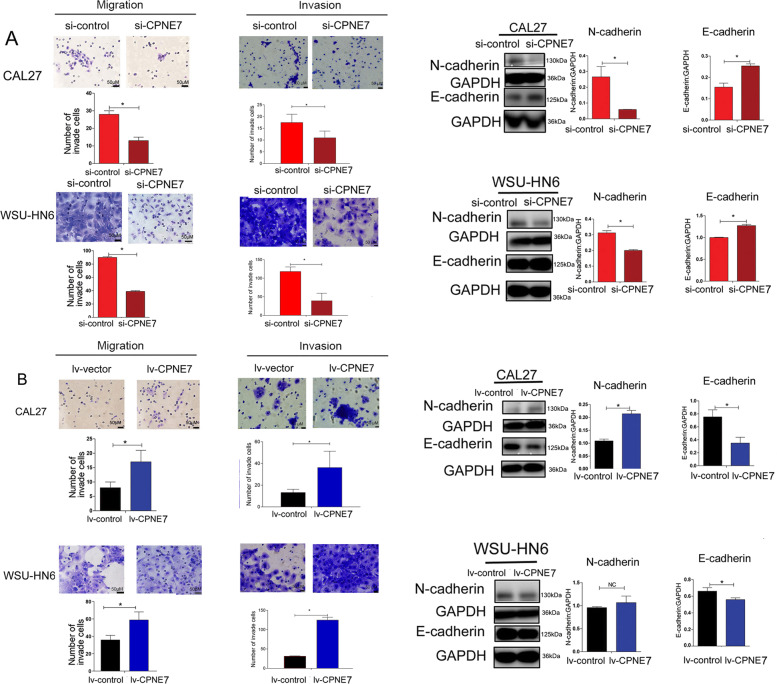
Overexpression and knockdown of CPNE7 in MSCs verified that CPNE7 promote metastasis of OSCC cell lines. **A** Knockdown of CPNE7 in OSCC-MSCs suppressed the migration of OSCC cell lines through EMT. **B** Overexpression of CPNE7 in OLK-MSCs promoted the migration of OSCC cell lines through EMT. The tests were repeated three times. Data were expressed as means ± SD. *p < 0.05.

To verify the regulatory effect of CPNE7 on the NF-κB pathway, we performed an MSC transfection assay to overexpress and knockdown CPNE7. We used siRNA to knockdown CPNE7 expression in OSCC-MSCs, and the treatment inhibited CAL27 and WSU-HN6 cell metastasis (Fig. 5A). In addition, the increase in E-cadherin and decrease in N-cadherin expression were determined by western blotting when CM was acquired from si-CPNE7-treated OSCC-MSCs co-cultured with CAL27 or WSU-HN6 (Fig. 5A).

A lentivirus was used to overexpress CPNE7 (lv-CPNE7) in OLK-MSCs. Transwell assays showed that CM obtained from OLK-MSCs treated with lv-CPNE7 significantly increased the metastatic ability of CAL27 and WSU-HN6 cells (Fig. 5B). Western blotting assays showed that the expression of N-cadherin was increased, with a simultaneous decrease in the expression of E-cadherin by CM of OLK-MSCs treated with lv-CPNE7 (Fig. 5B).

### CPNE7 interacts with Nucleolin,Iκßα and activeate NF-κB signalling pathway

**Fig. 6:**
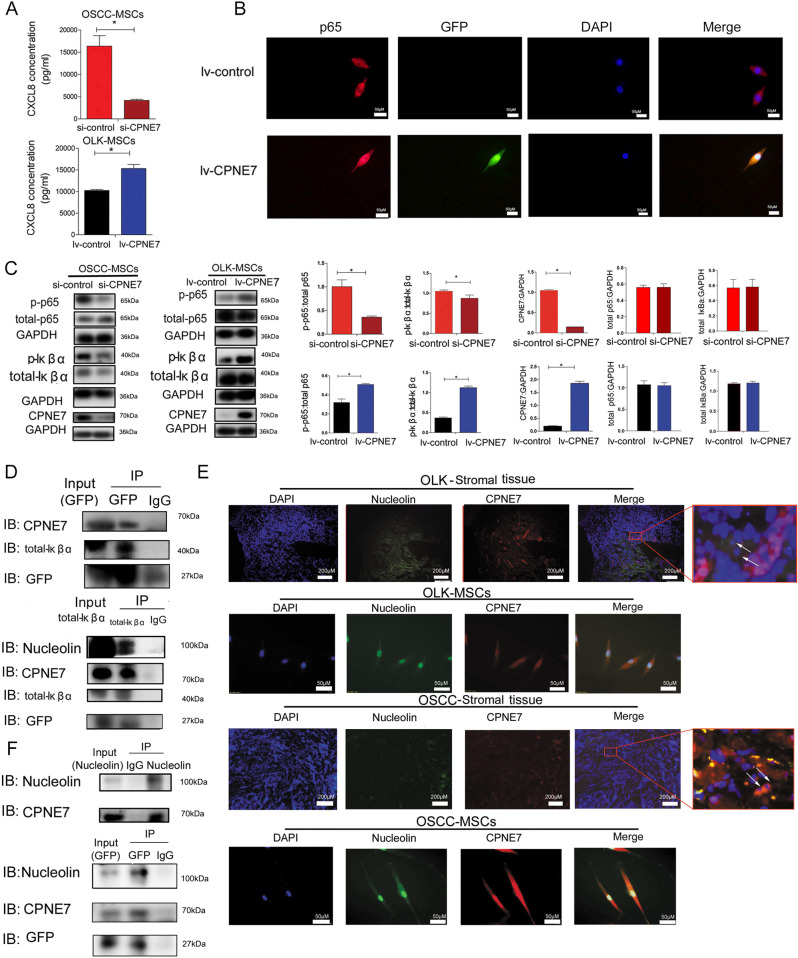
CPNE7 could enhance the secretion of CXCL8 and interact with Iκßα through the NF-κB pathway. **A** Overexpression and knockdown of CPNE7 in MSCs regulated CXCL8 secretion. **B** Immunofluorescence on overexpression of CPNE7 (GFP-tagged CPNE7) in OSCC-MSCs was performed to detect the localization of p65 (red) and GFP (green) were stained with DAPI (blue). **C** Western blot was used to measure the difference in the expression of p65 and IκBα (NF-κB pathway) in CPNE7-overexpressing OLK-MSCs and CPNE7-knockdown OSCC-MSCs. **D** Immunoprecipitated (IP) CPNE7 and whole-cell lysates (Input) were analysed by immunoblotting (IB) with anti- IκBα, Nucleolin, CPNE7, and GFP antibodies. **E** Co-localization of CPNE7 and Nucleolin in tissue and MSCs. The arrow indicated the co-expression of CPNE and Nucleolin in the same cells of the tissue. **F** Immunoprecipitated (IP) CPNE7 and whole-cell lysates (Input) were analysed by immunoblotting (IB) with anti- CPNE7, Nucleolin, and GFP antibodies. The tests were repeated three times. Data were expressed as means ± SD. *p < 0.05.

siRNA was used to knock down CPNE7 expression in OSCC-MSCs, CXCL8 level was significantly decreased in OSCC-MSCs treated with si-CPNE7 (Fig. 6A), and phosphorylated p65 and Iκßα decreased compared with those in the control (Fig. 6C).

Compared with lv-control, ELISA assay was used to measure the abundance of CXCL8 in the two groups, and the results showed that OLK-MSCs overexpressing CPNE7 (lv-CPNE7) could secrete more CXCL8 than the control cells (Fig. 6A). Immunofluorescence assay was performed showed that compared with lv-control, in the lentivirus-CPNE7-GFP (lv-CPNE7) the expression of p65 and GFP could was found in cytoplasm and nucleus. So, it speculated that CPNE7 could promote p65 released and translocated into the nucleus in OSCC-MSCS and p65 may be active in the NF-κB pathway (Fig. 6B). Furthermore, p65 and Iκßα phosphorylation were also increased in OLK-MSCs treated with lv-CPNE7 (Fig. 6C).

To determine the possible mechanisms of CPNE7 in the regulation of the NF-κB pathway, we predicted an interaction between CPNE7 and Iκßα. To confirm this speculation, a Co-IP assay was performed in OLK-MSCs treated with lv-CPNE7. In CPNE7-overexpressing OLK-MSCs, CPNE7 was fused with GFP protein. The cell lysate was incubated with IgG beads, Nucleolin, GFP, and Iκßα antibodies and the immunoprecipitated proteins were obtained. The two kinds of lysate assays showed that Nucleolin - GFP (CPNE7)- Iκßα could be pulled down (Figs. 6D, 6F). The above results suggest that CPNE7 could directly bind to Iκßα, Nucleolin.

In addition, we performed an immunofluorescence assay of tissue and MSCs with Nucleolin and CPNE7. The results showed that CPNE7 protein co-localized with Nucleolin protein in the cytomembrane (cytoplasm and nucleus) of stromal tissue and MSCs **(**Fig. 6E**)**. Next, we investigated the changing pattern of CPNE7 and Nucleolin in the progression from OLK-MSCs to OSCC-MSCs. Western blotting assays showed an increase in CPNE7 expression, whereas the expression of Nucleolin remained unchanged (Supplementary Fig. 4A). Consistent with the above results, no changes in Nucleolin were detected in OSCC-MSCs or OLK-MSCs treated with si-CPNE7 (Supplementary Fig. 4B, C). It speculated that Nucleolin may be the surface recpotor of CPNE7 [39], and combined with Iκßα to active NF-κB signalling pathway when they move into the cytoplasm.

## Discussion

**Fig. 7:**
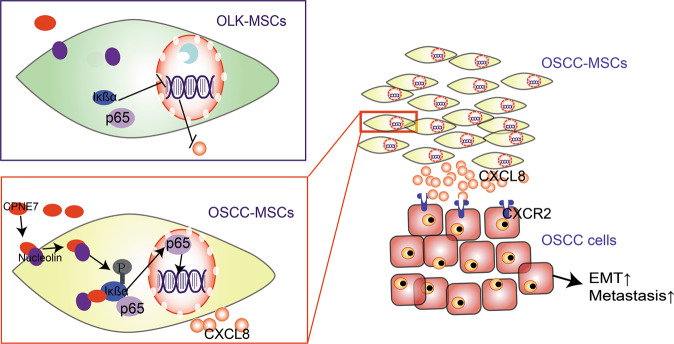
Schematic depiction. CPNE7 controls CXCL8 secretion through the NF-κB signalling pathway, promoting the metastasis of OSCC cells in the malignant transformation of OLK to OSCC.

The ability of MSCs to reprogram the TME plays a crucial role in cancer initiation and progression, and the existence of paracrine loops between MSCs and tumour cells has been suggested [10]. However, few studies have examined the role of MSCs in the development of OLK into OSCC. Here, we report that compared with OLK-MSCs, OSCC-MSCs can promote OSCC cell migration through EMT. In addition, CPNE7 bind to Nucleolin in cell surface and then conjugates with Iκßα when CPNE7 moved into cytoplasm. They could promote Iκßα phosphorylation and p65 translocate into nucleus activated the NF-κB pathway, which is closely associated with the metastasis of OSCC cells through CXCL8 secretion (Fig. 7).

Herein, this study showed that CAL27 and WSU-HN6 cells possessed stronger metastatic ability regulated by EMT when treated with OSCC-MSCs than OLK-MSCs. Similar results were shown not only in cell-cell indirect contact but also in the CM treatment method via the transwell system, which agreed with previous studies that tumour-derived (TME educated) MSCs could promote the metastasis of tumours through EMT [20, 40]. In the TME, the interaction between MSCs and tumours is indirect contact through soluble secreted factors [9, 10]. We observed that more CXCL8 was expressed and secreted by OSCC-MSCs to increase the metastatic ability of OSCC, which was also confirmed by the CXCL8 neutralizing antibody and CXCR2 inhibitor experiments. A study reported that CXCL8, a pro-inflammatory chemokine, plays an important role in metastasis by associating with tumour-related inflammation [27–30] and inducing the risk of OLK and OSCC, such as in the case of betel quid chewing and smoking [41, 42], but the regulatory pathways are still not reported to date. In our study, KEGG pathway analysis showed that CXCL8 was enriched in the nuclear factor-κB (NF-κB), the centre of some annotated signalling pathway [3], is essential for the initiation and maintenance of EMT [43]. Additionally, NF-κB signalling was active and the pathway inhibitor partially reduced the production of CXCL8 in the CM of OSCC-MSCs. Previous findings showing that tumour-derived MSCs secreted CXCL8 regulated by the NF-κB pathway were also confirmed in other tumours [31–33], and we extended these observations further by identifying the upstream regulator.

To study the regulator of the NF-κB pathway, RNA-sequencing was performed, which identified CPNE7 [36] associate with nodal metastasis status in the TCGA database. CPNE7, derived from the epithelium, is a ubiquitous calcium-dependent phospholipid-binding protein [44, 45]. First, our results showed that the expression of CPNE7 was increased in the connective tissue and MSCs of OSCC compared to OLK. CPNE7 was co-expressed with STRO-1 (one of the markers of MSCs [46, 47]) in the connective tissue of OLK and OSCC. Our results on the CPNE7 expression level were partly confirmed by tumour tissue data from the TCGA database (in comparisons between para- and tumour tissues). Second, we knocked down and overexpressed CPNE7 in MSCs and found that the CM could affect metastasis and E-cadherin and N-cadherin expression regulated by CXCL8 secretion. Previous findings showing that the copine family could modulated some key proteins of the NF-κB pathway [36, 45]. So, we speculated that CPNE7 is the regulator of the NF-κB pathway.

Finally, we investigated the regulatory mechanism involved in the CPNE7 activation of in the NF-κB signalling pathway. p65 is one of the most important transcription factors in the NF-κB pathway that assembles with and binds to the inhibitory IκBα to form a complex, which prevents the complex from translocating into the nucleus [43]. Copine family proteins contain two distinctive domains, a phospholipid-binding domain (C2 domains) of signalling proteins in the N-terminus and a protein interaction domain (von Willebrand factor type A domain, vWA) in the C-terminus [45, 48]. The underlying mechanism of CPNE7 regulation by the NF-κB pathway is poorly reported, but homologues of the copine family (such as copine I and copine III) [45] were reported to degrade IκB [36] and induce end protease processing of the N-terminus of p65 [43]. In our results, p65 was released and translocated into the nucleus in OSCC-MSCs and lv-CPNE7 and the expression of phosphorylated p65 and IκBα was higher in OSCC-MSCs than in OLK-MSCs (in lv-CPNE7 than in lv-control). So, it speculated that CPNE7 could promote p65 and IκBα phosphorylation and nuclear translocation of p65. In addition, Nuclelion, CPNE7, and IκBα were interacting with each other by Co-IP tests in our results. Therefore, we inferred that Nuclelion may be the cell surface receptor of CPNE7 CPNE7, Nuclelion-CPNE7-IκBα could promote IκBα phosphorylation and nuclear translocation of p65 and regulated the expression of CXCL8, but the detailed mechanism of downstream targeted genes and binding mode still needs to be explored in the future.

In conclusion, during the malignant transformation of OLK, MSCs induce a pro-metastatic phenotype, which is regulated by CXCL8 in the NF-κB pathway and controlled by the CPNE7-Nuclelion-IκBα complex. Targeted management with CPNE7 modulators in MSCs may represent a novel therapeutic approach for the prevention of the malignant transformation of OLK to OSCC.

## Supplementary information

Supplementary Files

STR Profile Report-CAL27

STR Profile Report-WSU-HN6

## Data Availability

Authors can confirm that all relevant data are included in the article and its supplementary information files. The datasets of RNA-sequencing in this study are available in the SRA data (PRJNA665945) (https://www.ncbi.nlm.nih.gov/sra).
