# Supplementary material for: Upregulation of CPNE7 in mesenchymal stromal cells promotes oral squamous cell carcinoma metastasis through the NF-κB pathway
Source: Cell Death Discov. 2021 Oct 14;7:294. doi: 10.1038/s41420-021-00684-w (PMC8516970; doi:10.1038/s41420-021-00684-w)
Supplement: Supplementary file 1 — Supplementary Files [file 41420_2021_684_MOESM1_ESM.pdf]

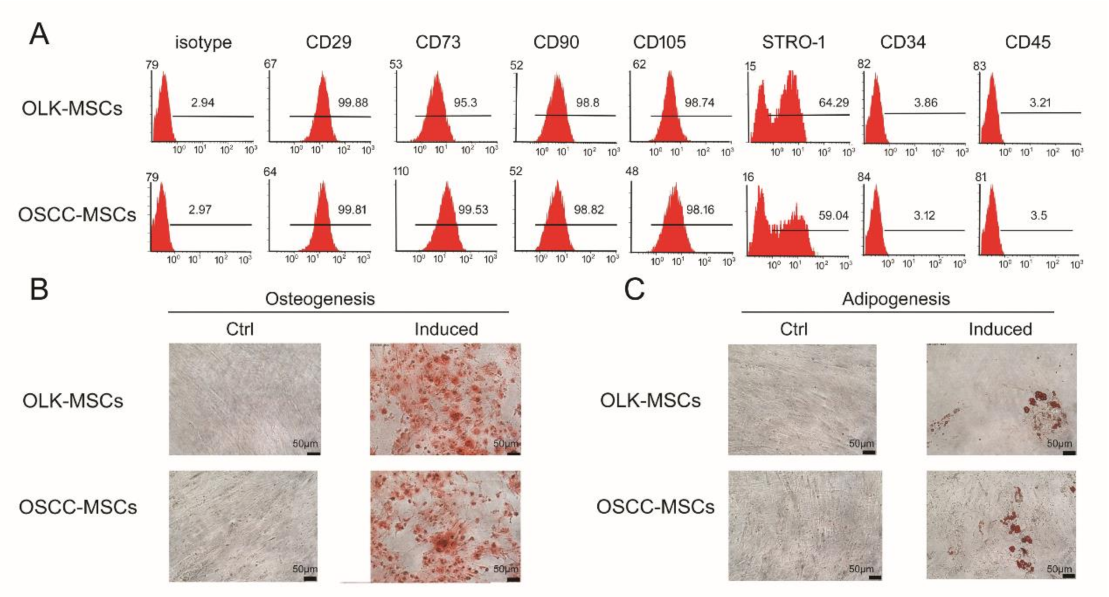

Supplementary Fig. 1 Identification of OLK-MSCs and OSCC-MSCs. A Analysis of MSC surface biomarkers (isotype control, CD29, CD73, CD90, CD105, STRO-1, CD34, CD45) in OLK-MSCs (n=3) and OSCC-MSCs (n=3). **B** Osteogenesis and adipogenesis of OLK-MSCs (n=3) and OLK-MSCs (n=3) (bar=50 μM). The passage of MSCs was 3 to 5.

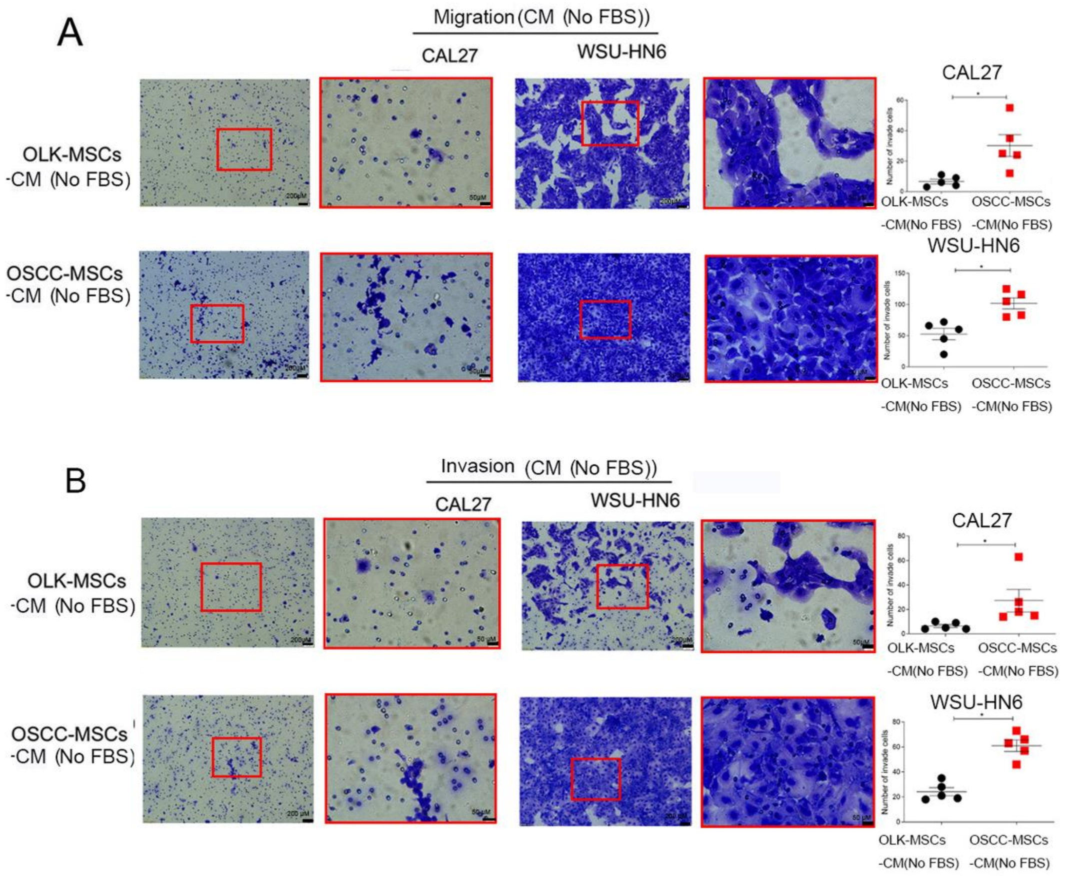

Supplementary Fig. 2 The conditioned medium of OSCC-MSCs without FBS promotes the metastasis of OSCC cell lines. The OSCC cell lines (CAL27 and WSU-HN6) were treated with

conditioned medium without FBS from OLK-MSCs (n=5) and OSCC-MSCs (n=5). The migration**(A)** and invasion**(B)** of tumour cell migration and invasion were detected. The tests were repeated three times. Data were expressed as means ± SD. *p<0.05.

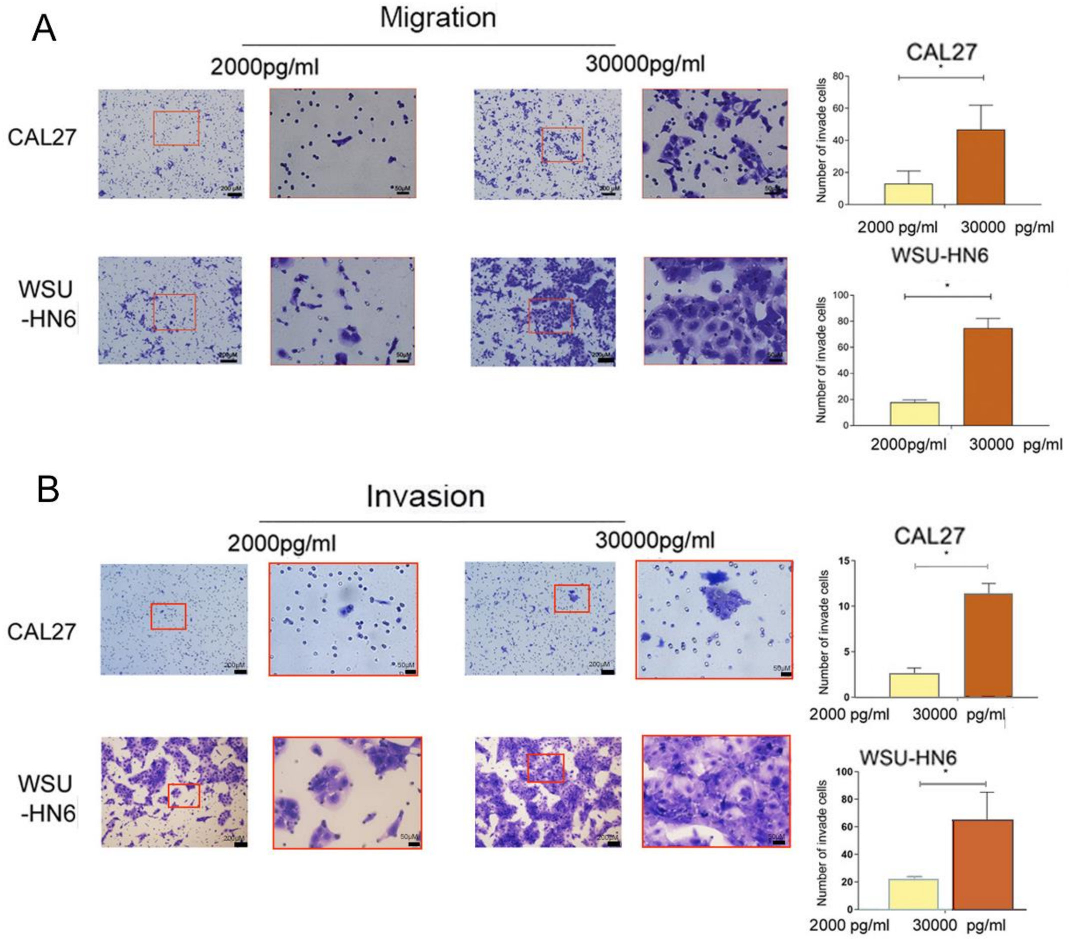

Supplementary Fig. 3 CXCL8 improves the metastasis of OSCC cell lines. Transwell assays were performed to detect tumor cell (CAL27 and WSU-HN6) migration and invasion mimics conditional medium from OLK-MSCs (the concertation of CXCL8 = 2000pg/ml) and OSCC-MSCs (the concertation of CXCL8 =30000 pg/ml). The tests were repeated three times. Data were expressed as means ± SD. *p<0.05.

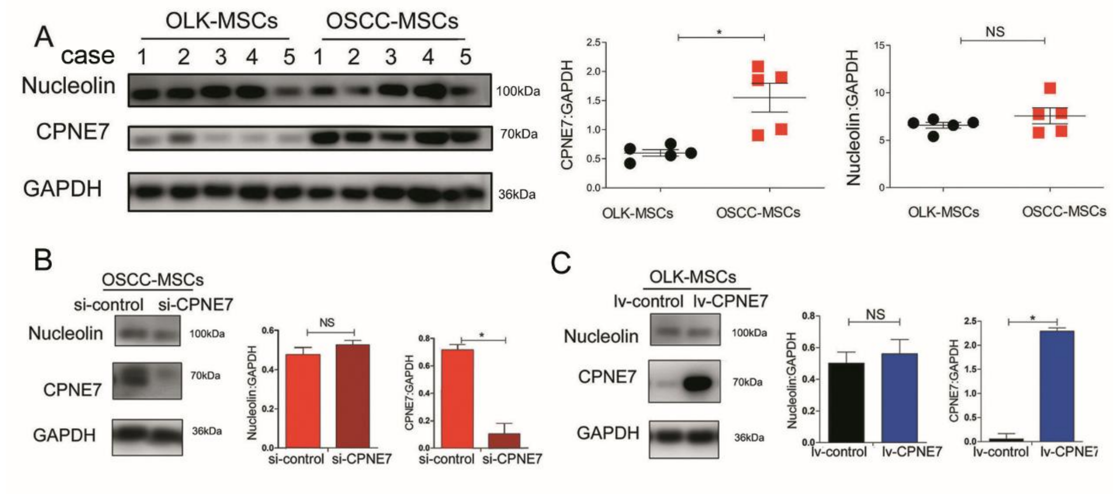

Supplementary Fig. 4 Western blotting was performed to detect the expression of CPNE7 and Nuclelion in OLK-MSCs (n=5) and OSCC-MSCs (n=5) (A), and CPNE7-overexpressing OLK-MSCs(B) and CPNE7-knockdown OSCC-MSCs(C).
