# Supplementary material for: Upregulation of CPNE7 in mesenchymal stromal cells promotes oral squamous cell carcinoma metastasis through the NF-κB pathway
Source: Cell Death Discov. 2021 Oct 14;7:294. doi: 10.1038/s41420-021-00684-w (PMC8516970; doi:10.1038/s41420-021-00684-w)
Supplement: Supplementary file 2 — STR Profile Report-CAL27 [file 41420_2021_684_MOESM2_ESM.pdf]

Cell Line Authentication Service
STR Profile Report

GENETIC TESTING BIOTECHNOLOGY

Sample Submitted By: Dr. Xiaoli Ji
Shandong University
Email Address: jixiaoli@email.sdu.edu.cn
Sales Order: 200509A
Cell Line Designation: CAL27
Date Sample Received: May 9th, 2020
Report Date: May 12th, 2020

Methodology: Nineteen short tandem repeat (STR) loci plus the gender determining locus, Amelogenin, were amplified using the commercially available EX20 Kit from AGCU. The cell line sample was processed using the ABI Prism® 3130 XL Genetic Analyzer. Data were analyzed using GeneMapper® ID v3.2 software (Applied Biosystems). Appropriate positive and negative controls were run and confirmed for each sample submitted.

Data Interpretation: Cell lines were authenticated using Short Tandem Repeat (STR) analysis as described in 2012 in ANSI Standard (ASN-0002) by the ATCC Standards Development Organization (SDO) and in Capes-Davis et al., Match criteria for human cell line authentication: Where do we draw the line? Int J Cancer. 2013;132(11):2510-9.

GTB™ performs STR Profiling following ISO 9001:2008 and ISO/IEC 17025:2005 quality standards.

There are no warranties with respect to the services or results supplied, express or implied, including, without limitation, any implied warranty of merchantability or fitness for a particular purpose. Genetic Testing Biotechnology (GTB) is not liable for any damages or injuries resulting from receipt and/or improper, inappropriate, negligent or other wrongful use of the test results supplied, and/or from misidentification, misrepresentation, or lack of accuracy of those results. Your exclusive remedy against GTB and those supplying materials used in the services for any losses or damage of any kind whatsoever, whether in contract, tort, or otherwise, shall be, at GTB's option, refund of the fee paid for such service or repeat of the service.

NOTE: According to the recommendations of *IJC* on cell line authentication, the report is valid for 3 years since the issue date.

Technical Questions?
GTB Technical Support
+86-512-67486171
service@jsdna.org
Section 505, Yixin BLD
SIP, Suzhou, 215123
Jiangsu, P.R. China

Ordering Questions?
order@jsdna.org
GTB Corporation
+86-512-62806339
Section 303, Yixin BLD
SIP, Suzhou, 215123
Jiangsu, P.R. China

GENETIC TESTING BIOTECHNOLOGY

Cell Line Authentication Service
STR Profile Report

Sales Order: 200509A

	Test Results for Submitted Sample		DSMZ Reference Database Profile	
**Loci**	**Query Profile:** CAL27		**Database Profile:** CAL27	
Amelogenin	X		X	
D3S1358	16			
D13S317	10	11	10	11
D7S820	10		10	
D16S539	11	12	11	12
Penta E	7			
TPOX	8		8	
TH01	6	9.3	6	9.3
D2S1338	23	24		
CSF1PO	10	12	10	12
Penta D	9	10		
D19S433	14	15.2		
vWA	14	17	14	17
D21S11	28	29		
D18S51	13			
D6S1043	12			
D8S1179	13	15		
D5S818	11	12	11	12
D12S391	18.3	20		
FGA	25			

The allele match algorithm compares the 8 core loci plus amelogenin only, even though alleles from all loci will be reported when available.

Note: Loci highlighted in grey (8 core STR loci plus Amelogenin) can be made public to verify cell identity. In order to protect the identity of the donor, ***please do not publish*** *the allele calls from all the STR loci tested.*

Explanation of Test Results

Cell lines with ≥80% match are considered to be related; i.e., derived from a common ancestry. Cell lines with between a 55% to 80% match require further profiling for authentication of relatedness.

- ☐ The submitted sample profile is human, but not a match for any profile in the DSMZ STR database.
- ☑ The submitted profile is an exact match for the following human cell line(s) in the DSMZ STR database (8 core loci plus Amelogenin): CAL27
- ☐ The submitted profile is similar to the following DSMZ human cell line(s):

e-Signature, Technician:

Digitally signed by Xuekun Chen
DN: cn=Xuekun Chen, o=Genetic Testing Biotechnology (Suzhou), ou=DNA Typing Section, email=order@jsdna.org, c=CN
Date: 2020.05.12 15:16:07 +08'00'

e-Signature, Reviewer:

Digitally signed by Xiankun Zhao
DN: cn=Xiankun Zhao, o=Genetic Testing Biotechnology (Suzhou), ou=Supervision Section, email=service@jsdna.org, c=CN
Date: 2020.05.12 17:00:51 +08'00'

GENETIC TESTING BIOTECHNOLOGY — GUARANTEE — CELL LINE AUTHENTICATION

More information

Addendum: Electropherogram for the customer's sample set 1 of 1

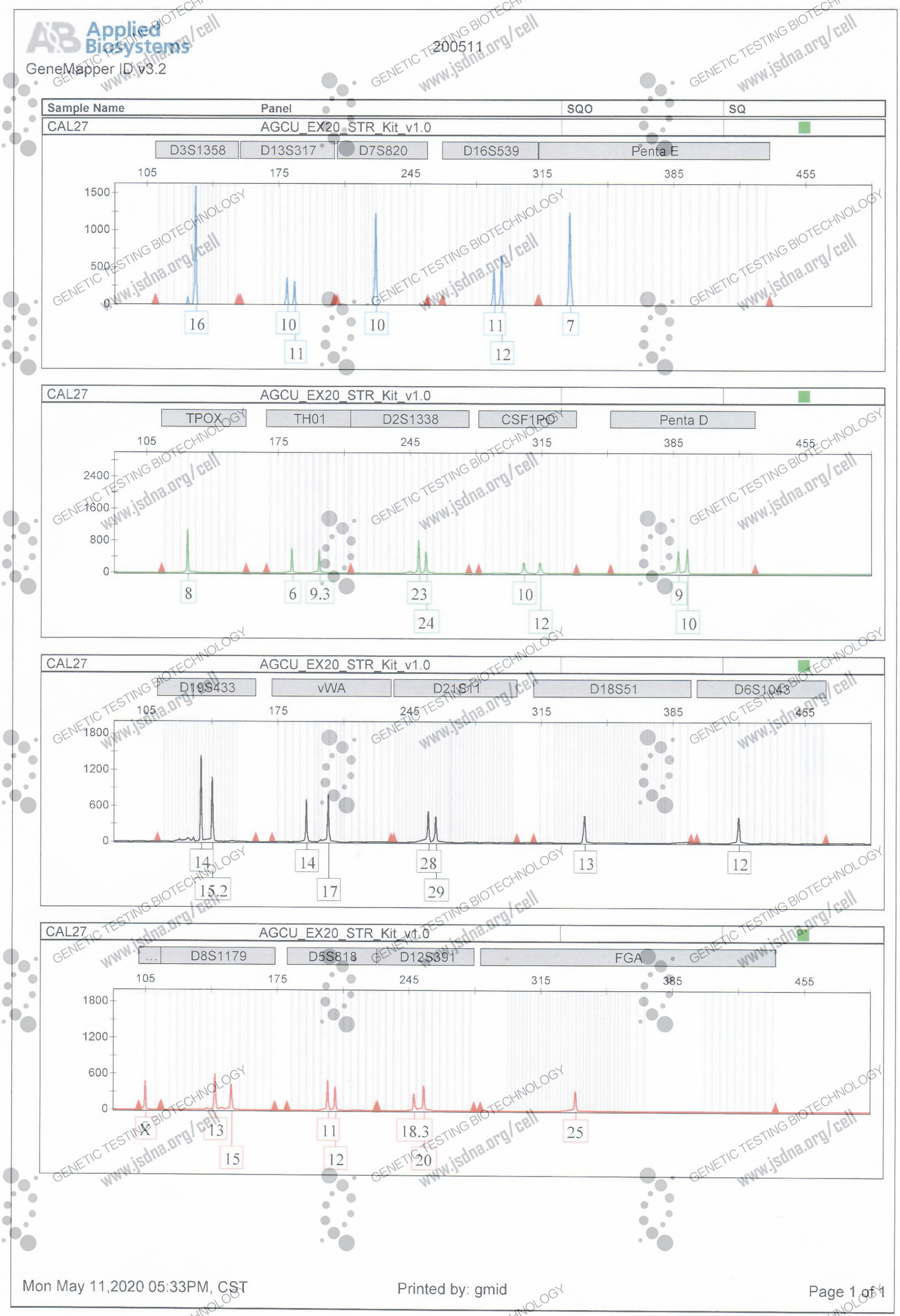

GENETIC TESTING BIOTECHNOLOGY www.jsdna.org/cell
