# Supplementary material for: Upregulation of CPNE7 in mesenchymal stromal cells promotes oral squamous cell carcinoma metastasis through the NF-κB pathway
Source: Cell Death Discov. 2021 Oct 14;7:294. doi: 10.1038/s41420-021-00684-w (PMC8516970; doi:10.1038/s41420-021-00684-w)
Supplement: Supplementary file 3 — STR Profile Report-WSU-HN6 [file 41420_2021_684_MOESM3_ESM.pdf]

GENETIC TESTING BIOTECHNOLOGY

Cell Line Authentication Service
STR Profile Report

**Sample Submitted By:**	Dr. Xiaoli Ji<br>Shandong University
**Email Address:**	jixiaoli@email.sdu.edu.cn
**Sales Order:**	200509C
**Cell Line Designation:**	WSU-HN6
**Date Sample Received:**	May 9th, 2020
**Report Date:**	May 12th, 2020

Methodology: Nineteen short tandem repeat (STR) loci plus the gender determining locus, Amelogenin, were amplified using the commercially available EX20 Kit from AGCU. The cell line sample was processed using the ABI Prism® 3130 XL Genetic Analyzer. Data were analyzed using GeneMapper® ID v3.2 software (Applied Biosystems). Appropriate positive and negative controls were run and confirmed for each sample submitted.

Data Interpretation: Cell lines were authenticated using Short Tandem Repeat (STR) analysis as described in 2012 in ANSI Standard (ASN-0002) by the ATCC Standards Development Organization (SDO) and in Capes-Davis et al., Match criteria for human cell line authentication: Where do we draw the line? Int J Cancer. 2013;132(11):2510-9.

GTB™ performs STR Profiling following ISO 9001:2008 and ISO/IEC 17025:2005 quality standards.

There are no warranties with respect to the services or results supplied, express or implied, including, without limitation, any implied warranty of merchantability or fitness for a particular purpose. Genetic Testing Biotechnology (GTB) is not liable for any damages or injuries resulting from receipt and/or improper, inappropriate, negligent or other wrongful use of the test results supplied, and/or from misidentification, misrepresentation, or lack of accuracy of those results. Your exclusive remedy against GTB and those supplying materials used in the services for any losses or damage of any kind whatsoever, whether in contract, tort, or otherwise, shall be, at GTB's option, refund of the fee paid for such service or repeat of the service.

NOTE: According to the recommendations of *IJC* on cell line authentication, the report is valid for 3 years since the issue date.

Technical Questions?
GTB Technical Support
+86-512-67486171
service@jsdna.org
Section 505, Yixin BLD
SIP, Suzhou, 215123
Jiangsu, P.R. China

Ordering Questions?
order@jsdna.org
GTB Corporation
+86-512-62806339
Section 303, Yixin BLD
SIP, Suzhou, 215123
Jiangsu, P.R. China

GENETIC TESTING BIOTECHNOLOGY

Cell Line Authentication Service
STR Profile Report

Sales Order: 200509C

	Test Results for Submitted Sample		ExPASy Reference Database Profile	
Loci	Query Profile: WSU-HN6		Database Profile: WSU-HN6	
Amelogenin	X		X	Y
D3S1358	18			
D13S317	13		13	
D7S820	10	11	10	11
D16S539	10	12	10	12
Penta E	8	14		
TPOX	8	10	8	10
TH01	6	9	6	9
D2S1338	17	25		
CSF1PO	11		11	
Penta D	9			
D19S433	11	14		
vWA	18	19	18	19
D21S11	32.2	33.2		
D18S51	13			
D6S1043	11	17		
D8S1179	13	14		
D5S818	11		11	
D12S391	18	19		
FGA	21	25		

The allele match algorithm compares the 8 core loci plus amelogenin only, even though alleles from all loci will be reported when available.

Note: Loci highlighted in grey (8 core STR loci plus Amelogenin) can be made public to verify cell identity. In order to protect the identity of the donor, ***please do not publish*** *the allele calls from all the STR loci tested.*

Explanation of Test Results

Cell lines with ≥80% match are considered to be related; i.e., derived from a common ancestry. Cell lines with between a 55% to 80% match require further profiling for authentication of relatedness.

☐ The submitted sample profile is human, but not a match for any profile in the ExPASy STR database.

☑ The submitted profile is an exact match for the following human cell line(s) in the ExPASy STR database (8 core loci): WSU-HN6

☐ The submitted profile is similar to the following ExPASy human cell line(s):

e-Signature, Technician:

Digitally signed by Xuekun Chen
DN: cn=Xuekun Chen, o=Genetic Testing Biotechnology (Suzhou), ou=DNA Typing Section, email=order@jsdna.org, c=CN
Date: 2020.05.12 16:44:58 +08'00'

e-Signature, Reviewer:

Digitally signed by Xiankun Zhao
DN: cn=Xiankun Zhao, o=Genetic Testing Biotechnology (Suzhou), ou=Supervision Section, email=service@jsdna.org, c=CN
Date: 2020.05.12 17:02:36 +08'00'

GENETIC TESTING BIOTECHNOLOGY — GUARANTEE — CELL LINE AUTHENTICATION

More information

Addendum: Electropherogram for the customer's sample set 1 of 1

GENETIC TESTING BIOTECHNOLOGY

Cell Line Authentication Service
STR Profile Report

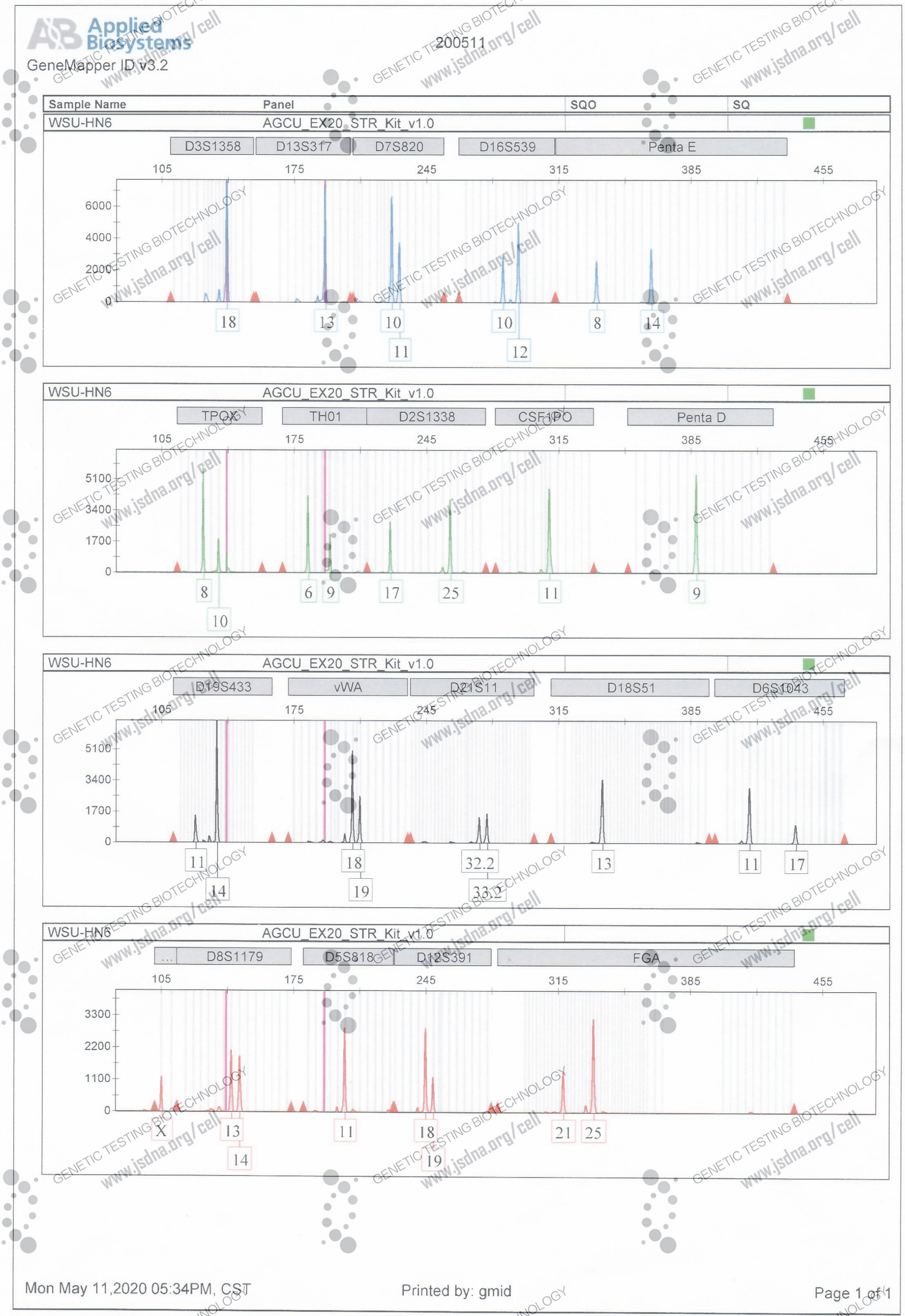

GENETIC TESTING BIOTECHNOLOGY www.jsdna.org/cell
